# Changing prescribing behaviours with educational outreach: an overview of evidence and practice

**DOI:** 10.1186/s12909-019-1735-3

**Published:** 2019-08-14

**Authors:** Breanne E. Kunstler, Alyse Lennox, Peter Bragge

**Affiliations:** BehaviourWorks Australia, Monash Sustainable Development Institute, Monash University, 8 Scenic Boulevard, Clayton, Melbourne, VIC 3800 Australia

**Keywords:** Education, Academic detailing, Educational outreach, General practice, Primary care, Inappropriate prescribing, Healthcare

## Abstract

**Background:**

General practitioners (GPs), or family practitioners, are tasked with prescribing medications that can be harmful to the community if they are inappropriately prescribed or used (e.g. opioids). Educational programs, such as educational outreach (EO), are designed to change the behaviour of health professionals. The purpose of this study was to identify the efficacy of EO programs at changing the prescribing behaviour of GPs.

**Methods:**

This study included an evidence and practice review, comprising a rapid review supplemented by interviews with people who are familiar with EO implementation for regulation purposes. Seven databases were searched using terms related to health professionals and prescribing. Systematic and narrative reviews published in English after 2007 were included. Non-statistical analysis was used to report intervention efficacy. Three government representatives participated in semi-structured interviews to aid in understanding the relevance of review findings to the Victorian context. Interviews were transcribed verbatim and thematically analysed for emerging themes.

**Results:**

Fourteen reviews were identified for the evidence review. Isolated (e.g. EO program delivered by itself) and multifaceted (e.g. EO program supplemented by other interventions) programs were found to change prescribing behaviours. However, limited evidence suggests that EO can successfully change prescribing behaviours specific to GPs. Isolated EO can successfully change health professional prescribing behaviours, although cheaper alternatives such as letters might be just as effective. Multifaceted EO can also successfully change health professional prescribing behaviours, especially in older adults, but it remains unclear as to what combination of interventions works best. Success factors for EO reported by government representatives included programs having practical rather than didactic foci; making EO compulsory; focussing EO on preventing adverse events; using monetary or professional development incentives; and in-person delivery.

**Conclusions:**

Educational outreach can successfully change prescribing behaviours but evidence specific to GPs is lacking. Key characteristics of EO that could optimise success include ensuring the EO program is tailored, involves practical learning and uses incentives that are meaningful to clinicians.

**Electronic supplementary material:**

The online version of this article (10.1186/s12909-019-1735-3) contains supplementary material, which is available to authorized users.

## Background

Prescription medications are a cornerstone of community-based healthcare, especially in first world countries. They are used to successfully manage a broad range of health conditions that appear in general medical practices including bacterial infections, chronic conditions such as diabetes, mental health conditions and everyday needs such as contraception. However, some classes of prescription medications (e.g. opioids) carry risk to the community if their prescription or use is inappropriate [1].

Inappropriate prescribing includes a variety of potentially harmful prescribing practices such as inappropriate dosage and prescribing medications that interact with others or lead to adverse events [2]. Inappropriate prescribing of medications, especially those that are opioid-based analgesics and psychoactive, can be harmful and even fatal to patients by potentially facilitating inappropriate use.

Inappropriate use of prescription medications is a growing problem globally. The number of deaths in the US involving opioids has increased from just over 10,000 to 49,068 in 15 years (2002–2017) [3]. In Australia, medication-induced deaths (i.e. the death was directly attributed to medication use) have steadily increased since 2011 where 7.5 deaths were recorded per 100,000 people in 2016, with most of these deaths being accidental [4]. In 2016, the typical picture of an Australian dying from a medication-induced death was a middle-aged male who was accidentally misusing prescription medications (e.g. benzodiazepines or oxycodone) together with several other medications (polypharmacy) [4].

Community-based general medical practitioners (known in Australia as general practitioners [GPs]) are one professional group who are well placed to identify people who have the potential to inappropriately use prescription medications. Education and other support strategies provided to GPs and other health professionals could help to reduce inappropriate prescribing to this group. One example of such strategies is educational outreach (EO), also referred to in the literature as ‘academic detailing’. Educational outreach involves a trained facilitator delivering a face-to-face program in a health professional’s setting (e.g. GP clinic) with the aim to change clinician behaviour, such as prescribing behaviours [5]. Educational outreach programs can focus largely on education (e.g. an educational workshop delivered as part of an isolated EO program that includes education about an issue and ways to address it) or include a variety of supplemental or additional strategies like providing reminder letters or audit and feedback (i.e. multifaceted EO). Educational outreach programs can vary regarding the participants involved, the type of content delivered, the way it is delivered and the outcomes achieved [6].

There is review-level evidence supporting use of EO for changing prescribing behaviours [7]. However, this review was published in 2001 and, therefore, requires updating. Thus, the aim of this evidence and practice review was to identify recent literature and examine the efficacy of EO at changing prescribing behaviours (e.g. reducing inappropriate prescribing) of health professionals and, more specifically, GPs. Furthermore, this review aims to report factors that are perceived to facilitate GP engagement in EO.

## Methods

This evidence and practice review has two components; a rapid review of existing literature supplemented by a practice review including interviews with people who are familiar with EO implementation for regulation purposes. The reporting of this study was informed by PRISMA [8] and Standards for Reporting Qualitative Research [9] protocols.

### Evidence review methods

A rapid review methodology was employed for this study. Rapid reviews are an emerging method of evidence synthesis that differ from systematic reviews primarily by timeframe (e.g. rapid reviews can take 6–10 weeks to produce, compared to systematic reviews that can take up to 2 years) and included study types (e.g. rapid reviews synthesise evidence mainly from existing systematic reviews, whereas systematic reviews often include all study types) [10]. The increased interest in rapid reviews over recent years has encouraged the development of the Cochrane Rapid Reviews Methods Group, which is charged with refining and informing rapid review methodology [11].

Rapid reviews are valuable to government policy officers due to the need to quickly review existing evidence to answer pertinent questions that inform rapidly developing and changing policy directions [12–14]. Although rapid reviews focus on synthesised evidence as the unit of analysis, important methodological components of traditional systematic reviews remain, such as the use of systematic and comprehensive search strategies across multiple academic databases [14]. Rapid reviews have been reported as having similar conclusions to systematic reviews on the same topic [15].

#### Protocol and registration

A review protocol was established a priori and retrospectively registered on December 12, 2018 (PROSPERO ID: CRD42018115742).

#### Eligibility criteria

Systematic and narrative reviews were considered for inclusion if they met the following criteria:

1. Included primary studies of GPs or a targeted group of health practitioners in primary care or community settings;
2. Included primary studies that examined the efficacy of EO or academic detailing programs, defined as programs that involve visits from a trained facilitator to the health professional in their own setting (e.g. GP clinic) to provide a face-to-face program with the aim to change their behaviour (e.g. prescribing behaviour). Programs could include a variety of components, but one component must have been educational; and
3. Published after 2007 to ensure recency.

#### Information sources

A comprehensive search using a variety of keywords and databases and restricted to the last 10 years (2008–2018) and English language was conducted in May 2018. Seven databases were searched to identify relevant reviews, including: EMBASE, Cochrane Database of Systematic Reviews, Cochrane Database of Abstracts of Reviews of Effects, MEDLINE, PsycINFO, CINAHL and International Pharmaceutical Abstracts. Publications by the Cochrane Effective Practice and Organisation of Care (EPOC) were searched separately on their website. These sources were chosen for their large size and relevant disciplinary foci.

The search terms used to search the databases included combinations of key words (and associated synonyms) that belonged to three categories, including:

- Population: doctor, general practitioner, family doctor, health professional.
- Intervention: educational outreach, academic detailing, knowledge translation.
- Outcome: prescribing, quality assurance, safe, opioid.

Key words were entered into an appropriate syntax for each individual database and combined with appropriate wildcards (Additional file 1). Subject terms, such as ‘general practitioners’, were also used to narrow the database search to the population of interest if the database provided this functionality. Forwards and backwards citation screening were also completed using all the included papers to ensure as many systematic reviews on this topic were identified as possible. Database and Google scholar alerts were established to ensure reviews published after searches were completed were also found. No new relevant publications were identified by 15 December 2018, when these alerts were ceased.

#### Study selection

All articles were uploaded to Covidence, an online software system, for duplicate title, abstract and full text screening. All conflicts were resolved via consensus and a third reviewer if appropriate.

#### Data extraction and synthesis

One reviewer performed the data extraction and quality appraisal. Data extracted from relevant reviews included: author name/s, date published, study design, study aim,

participants, methods, authors’ findings and relevance of the findings to the research question.

#### Risk of bias

A Measurement Tool to Assess Systematic Reviews 2 (AMSTAR 2) is a valid tool that is used to appraise the quality of systematic reviews and to establish the level of confidence one should have in the findings from appraised reviews [16]. The AMSTAR 2 was used to perform duplicate risk of bias assessment for all systematic reviews, which informed the overall interpretation of the available evidence base. Reviews were categorised into low risk of bias (> 61%), moderate risk of bias (31–60%) and high risk of bias (< 31%).

### Practice review methods

One-on-one semi-structured interviews were conducted to allow in-depth discussions of EO provision and the success factors tested by government-employed EO providers vested in appropriated prescribing in Victoria, Australia.

#### Ethics

Ethics approval was obtained from the Monash University Human Research Ethics Committee [Reference: 2018–13,773-19,116] prior to data collection commencing. All interview participants provided written consent prior to participation.

#### Researcher characteristics and context

Two co-authors trained in qualitative methodology conducted the interviews. They were both involved in all aspects of the study design, including interview guide design, recruitment and analysis. Their roles as full-time researchers contrasted with the qualifications of the interviewees, who primarily worked in the public service. Thus, the qualifications and experiences of the interviewers were largely detached from those of the interviewees.

#### Sampling strategy

Participants were identified through professional networks and were purposively selected based upon their experience and/or expertise in EO for safe prescribing in the regulatory setting [17].

#### Data collection

Semi-structured interviews were performed over the telephone during June 2018 using an interview guide (Additional file 2) that was reviewed and refined prior to data collection. All interviews were audio recorded.

#### Units of study

Representatives of government pharmacy and health professional regulation bodies, as well as providers of EO to GPs, were approached to participate in interviews.

#### Data processing and analysis

Interview audio-recordings were transcribed verbatim, proofread for accuracy and kept on a password protected database. All transcripts were read several times to establish data familiarity and uploaded to NVivo for organising (NVivo10, QSR International Pty Ltd. 2014). Interview transcripts were coded, and all codes were used to identify emergent themes. Direct quotations from interview transcripts were used to illustrate emergent themes. Participant identifiers (i.e. role and responsibilities) were de-identified.

#### Techniques to enhance trustworthiness

The exclusion of the co-author who led the evidence review from the practice review, as well as using interviewers who did not work in the field of EO or policy, reduced the likelihood of social desirability bias, or the desire of interviewees to appear favourable to the interviewer [18].

## Results

### Evidence review results

#### Study selection

**Fig. 1:**
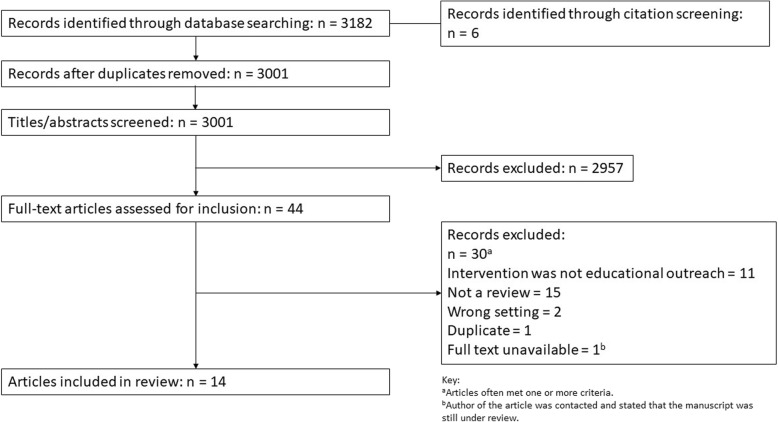
PRISMA diagram of screening and selection

Overall, 3182 citations were obtained from database searching and database alerts (Fig. 1). An additional six citations were also identified via forwards and backwards citation screening of relevant systematic reviews. After duplicate title and abstract screening, 44 citations moved through to the full text screening stage and 14 reviews were included, comprising 13 systematic reviews and one narrative review [19].

#### Study characteristics

The included systematic reviews collectively reviewed 707 studies (Table 1). Two reviews focused on GPs or family physicians [2, 23] and the remaining reviews expanded their scope to include other health professionals and prescribers. Only one review was entirely focused on the efficacy of EO on prescribing by GPs [23].

**Table 1:** Characteristics of included systematic reviews

Author (year)	Aim	Population	Number of studies	Intervention of focus	Outcome measured	Author’s conclusions	Quality appraisal^b^
Alagoz et al. (2018) [20]	Identify the efficacy of external change agents^a^ on organisational change in health care.	Staff in primary care clinics and general practices.	21 studies (20 cluster RCTs, 1 RCT)	External change agents	Practice level change	Thirteen of 21 multifaceted interventions that included at least two components (from EO, educational materials, audit and feedback, coaching [practice facilitation] and system support) were efficacious at changing practice behaviour. Practice facilitation, or individualised follow-up coaching, was an important component of successful interventions.	9/13
Baker et al. (2015) [21]	Compare the efficacy of tailored interventions vs. non- tailored interventions (e.g. EO programs) at improving professional practice and health outcomes.	Health professionals	32 cluster RCTs	Tailored interventions vs. non- tailored interventions (e.g. EO programs)	Implementation of recommended practice (e.g. following prescribing guidelines)	Tailored interventions can be more efficacious than non-tailored interventions, but the effect tends to be small to moderate. Due to the small number of studies, the authors remain uncertain if there is a true difference in the efficacy of the interventions.	12/16
Chauhan et al. (2017) [22]	Establish the efficacy and feasibility of behaviour change interventions in primary health care settings on patient and professional outcomes.	GPs, nurses, midwives, physician assistants, pharmacists, social workers, psychologists and dieticians who primarily manage patients with chronic disease.	138 systematic reviews (3502 individual studies)	Behaviour change interventions	Health professional behaviour change	Interventions that include enablement, education and training delivered in the context of collaborative teamwork can change the behaviour of health professionals working in primary care.	11/12
Chhina et al. (2013) [23]	To report the efficacy EO (as a stand-alone intervention) has on prescription behaviour in primary care	Family physicians (GPs)	15 studies (11 RCTs, 4 observational)	Educational outreach as a stand-alone intervention	Prescription rates of various medications	Educational outreach, as a stand-alone intervention, was moderately efficacious at changing prescribing behaviour of family physicians. Few studies examined regulated medications, such as benzodiazepines, and these studies reported inconsistent findings.	8/13
Clyne et al. (2016) [2]	Establish the efficacy of interventions aimed at reducing PIP of medications to older adults in the community	GPs	12 RCTs	Academic detailing delivered as part of a multifaceted intervention	Rates of PIP	Multifaceted interventions including academic detailing modestly reduced PIP in older adults. However, only three studies contributed to this finding.	8/13
Forsetlund, Eike, Gjerberg, and Vist (2011) [24]	Identify the efficacy of interventions that intend to reduce PIP in care homes	Prescribers in nursing homes	20 RCTs	Not limited to any intervention	Use of or prescribing of medications	Educational interventions (e.g. isolated or multifaceted EO, educational meetings) can reduce inappropriate medication use. However, it is unclear which of these interventions are more efficacious due to poor quality evidence.	9/13
Green, Taylor, and Torgerson (2012) [25]	Identify the educational interventions that can improve prescribing behaviours.	Doctors, medical students and health professionals.	187 systematic reviews (unclear number of primary studies)	Medical education at all levels	Health professional behaviour	Active educational strategies (e.g. EO) appeared more efficacious at changing behaviour than passive strategies (e.g. giving an information leaflet).	8/12
Johnson and May (2015) [26]	Identify the components of successful behaviour change interventions targeted towards professional practice behaviours.	Health professionals in primary and secondary care.	67 systematic reviews (unclear number of individual studies)	Professional behaviour change interventions	Professional practice behaviours	Interventions that include normative restructuring, relational restructuring, modifying peer group norms via programs like EO, emphasising expectations of external groups (e.g. via audit and feedback) might successfully change professional practice behaviours.	9/12
Kamarudin, Penm, Chaar, and Moles (2013) [27]	Identify educational interventions and methods that can improve prescribing behaviour	Medical (e.g. GPs) and non-medical prescribers	47 studies (20 RCTs, 15 non-RCTs, 12 before-after)	Educational outreach	Inappropriate prescribing	Educational outreach can successfully reduce inappropriate prescribing of benzodiazepines and dietary supplements. Heterogeneity between studies limits ability to draw confident conclusions.	8/13
Loganathan, Singh, Franklin, Bottle, and Majeed (2011) [28]	Establish the efficacy of interventions aimed at reducing inappropriate prescribing in care homes.	Health professionals prescribing medications to older adults	16 studies (11 cluster RCTs, 3 before-and-after, 2 RCTs)	Interventions to reduce inappropriate prescribing.	Inappropriate prescribing	There is no current intervention that is efficacious at improving prescribing in care homes. However, education has shown the most promise, especially when delivered in an interactive way (e.g. workshops) with more than one health professional (e.g. physicians and nurses) and followed-up.	7/13
O’Brien et al. (2008) [6]	Identify the efficacy of EO visits on health professional practice and patient outcomes	Health professionals	69 RCTs	Educational outreach	Professional performance (e.g. prescribing behaviours) and healthcare outcomes	Educational outreach visits had a smaller, but more consistent, effect on prescribing behaviours compared to other behaviours (e.g. cardiovascular screening). Educational outreach visits delivered alone or with other interventions (e.g. reminders) have small effects on prescribing behaviour.	12/16
Ostini et al. (2009) [29]	Establish the efficacy of different interventions on supporting the adoption of safe, appropriate and/or cost- effective prescribing.	Health professionals prescribing medications outside of the hospital inpatient setting.	29 studies (22 RCTs, 4 controlled before-and-after, 3 controlled clinical trials)	Not limited to any intervention	Safe, appropriate and/or cost-effective prescribing	Educational outreach and audit and feedback interventions were most researched and show positive results for changing prescribing behaviours.	5/13
Smith and Tett (2010) [10]	Identify interventions used to improve the prescribing of benzodiazepines	Health professionals	32 studies (16 RCTs, 4 controlled trials, 2 observational, 2 convenience sample, 3 cohort, 1 randomised trial, 2 quasi-experimental)	Not limited to any intervention	Inappropriate/appropriate prescribing of benzodiazepines	Multifaceted interventions might be more successful than isolated education interventions at reducing benzodiazepine prescribing.	N/A
Thompson Coon et al. (2014) [30]	Establish the efficacy of interventions used to reduce inappropriate prescribing of antipsychotics to older adults who have dementia and reside in care.	Health professionals	22 studies (11 before-and-after, 6 RCTs, 5 controlled clinical trials)	Not limited to any intervention	Change in use of antipsychotics	Interventions to reduce inappropriate prescribing, such as educational outreach, might work in the short term.	8/13

Notes

^a^External change agents are defined by Alagoz et al., 2018 as people external to the primary care clinic who influence the practices of the clinic in a desirable way

^b^The overall score is calculated from all items that were applicable to the study. For detail, see Table 2

*EO* educational outreach, *GP* general practitioner, *PIP* potentially inappropriate prescribing, *RCT* randomised controlled trial, *RT* randomised trial

#### Risk of bias within and across studies

Thirteen reviews (comprising three reviews of reviews and 10 systematic reviews of primary studies) were graded following the AMSTAR 2 guidelines [16]. The narrative review [19] was not graded using AMSTAR 2 as this tool is not designed to appraise non-systematic reviews. All three reviews of reviews satisfied most applicable AMSTAR 2 criteria [22, 25, 26]. Of the 10 systematic reviews, only one [29] satisfied less than half of the applicable AMSTAR 2 criteria. This indicates that overall, risk of bias of these reviews was low to moderate (Table 2).

**Table 2:** Risk of bias appraisal of included systematic reviews

Criterion (AMSTAR 2)	Alagoz et al. (2018) [20]	Baker et al. (2015) [21]	Chauhan et al. (2017) [22]	Chhina et al. (2013) [23]	Clyne et al. (2016) [2]	Forsetlund et al. (2011) [24]	Green et al. (2012) [25]	Johnson and May (2015) [26]	Kamarudin et al. (2013) [27]	Loganathan et al. (2011) [28]	O’Brien et al. (2008) [6]	Ostini et al. (2009) [29]	Smith et al., (2010) [19]	Thompson Coon et al. (2014) [30]
1. Did the research questions and inclusion criteria for the review include the components of PICO?	Yes	Yes	Yes	Yes	Yes	Yes	Yes	Yes	Yes	Yes	Yes	No	–	Yes
2. Did the report of the review contain an explicit statement that the review methods were established prior to the conduct of the review and did the report justify any significant deviations from the protocol?	No	No	Yes	No	No	No	Yes	Yes	No	No	No	No	–	Yes
3. Did the review authors explain their selection of study designs for inclusion in the review?	No	No	No	No	No	No	No	No	No	No	No	No	–	No
4. Did the review authors use a comprehensive literature search strategy?	Partial yes	Yes	Partial yes	Partial yes	Partial yes	Partial Yes	Partial yes	Partial yes	Partial yes	Partial yes	Partial yes	Partial yes	–	Partial yes
5. Did the review authors perform the study selection in duplicate?	Yes	Yes	Yes	Yes	Yes	Yes	Yes	Yes	Yes	Yes	Yes	Yes	–	Yes
6. Did the review authors perform data extraction in duplicate?	No	Yes	Yes	Yes	No	No	No	No	Yes	No	Yes	Yes	–	No
7. Did the review authors provide a list of excluded studies and justify the exclusion?	Partial yes	Yes	No	No	No	Partial Yes	No	No	No	No	Yes	No	–	No
8. Did the review authors describe the included studies in adequate detail?	Partial yes	Yes	Partial yes	Partial yes	Yes	Partial Yes	Partial yes	Partial yes	No	Partial yes	Partial yes	No	–	Partial yes
9. Did the review authors use a satisfactory technique for assessing the risk of bias in individual studies that were included in the review?	Yes	Yes	Yes	Partial yes	Yes	Partial Yes	Yes	Yes	No	Partial yes	Partial yes	Unclear	–	No
10. Did the review authors report on the sources of funding for the studies included in the review?	No	No	Yes	No	Yes	No	No	No	Yes	No	No	No	–	No
11. If meta-analysis was performed, did the review authors use appropriate methods for statistical combination of results?	N/A	Yes	N/A	N/A	N/A	N/A	N/A	N/A	N/A	N/A	Yes	N/A	–	N/A
12. If meta-analysis was performed, did the review authors assess the potential impact of risk of bias in individual studies on the results of the meta-analyses or other evidence synthesis?	N/A	Yes	N/A	N/A	N/A	N/A	N/A	N/A	N/A	N/A	Yes	N/A	–	N/A
13. Did the authors account for risk of bias in individual studies when interpreting/discussing the results of the review?	Yes	Yes	Yes	No	Yes	Yes	Yes	Yes	Yes	Yes	Yes	Yes	–	Yes
14. Did the review authors provide a satisfactory explanation for and discussion of heterogeneity observed in the results of the review?	Yes	Yes	Yes	Yes	Yes	Yes	Yes	Yes	Yes	Yes	Yes	Yes	–	Yes
15. If they performed quantitative synthesis, did the review authors carry out an adequate investigation of publication bias (small study bias and discuss its likely impact on the results of the review)?	N/A	No	N/A	N/A	N/A	N/A	N/A	N/A	N/A	N/A	No	N/A	–	N/A
16. Did the review authors report any potential sources of conflict of interest, including any funding they received for conducting the review?	Yes	Yes	Yes	Yes	No	Yes	No	Yes	Yes	No	Yes	No	–	Yes
TOTAL yes / applicable items (%)	9/13 (69%)	12/16 (75%)	11/12 (92%)	8/13 (62%)	8/13 (62%)	9/13 (69%)	8/12 (66%)	9/12 (75%)	8/13 (62%)	7/13 (54%)	12/16 (75%)	5/13 (38%)	–	8/13 (62%)

All systematic reviews performed well in the areas of discussing heterogeneity between studies and duplicating study selection. Most studies satisfactorily described inclusion criteria (11/13). Conversely, few (6/13) systematic reviews undertook duplicate data extraction or provided a list of excluded studies (4/13).

#### Results synthesis

The following results section includes a synthesis of the findings from 13 systematic reviews and one narrative review, with emphasis placed on the efficacy of EO in isolation or as part of multifaceted programs aimed at changing health professional prescribing behaviour.

The included reviews focused more on the efficacy of multifaceted EO programs compared to isolated EO programs. Most of the reviews included in the following synthesis examined the efficacy of EO at changing the prescribing behaviour of various health professionals, with minimal reference made specifically to GPs.

##### Can EO be used to successfully change the prescribing behaviour of GPs?

Two systematic reviews specifically examined the efficacy EO for changing GP prescribing behaviour [2, 23].

Clyne et al. [2], by reviewing 12 studies (156,529 older adults), concluded that multifaceted interventions including EO can reduce inappropriate prescribing by GPs to older adults. Although most results were small, the effect of EO appeared larger where baseline inappropriate prescribing rates were high [2].

Chhina et al. [23] reviewed 15 studies and reported that EO was efficacious at changing prescribing behaviours of family physicians (GPs), including reductions in inappropriate prescribing. The medication classes being prescribed were not similar between studies, which had different outcomes, suggesting that the efficacy of EO at reducing inappropriate prescribing by GPs might change if a specific focus was to be placed on a certain medication class (e.g. benzodiazepines compared to antibiotics).

Findings from primary studies that reported the efficacy of EO at changing prescribing behaviours of GPs that were identified or referred to by the included reviews are outlined in Table 3. Overall, the key findings from these primary studies, together with the findings from two systematic reviews [2, 23], suggest that EO as both an isolated and multifaceted intervention shows promise for changing prescribing behaviours of GPs.

**Table 3:** Key findings from primary studies specific to GPs, prescribing and EO included in all systematic reviews

Author (date)^a^: Study design (parent review/s)	Behaviour	Intervention	Key findings
Atkin, Ogle, Finnegan, and Shenfield (1996): RCT (Chhina et al., 2013) [23]	Concurrent medication prescription to older adults	EO visit including education on adverse medication reactions and the importance of hospital-admission prevention in older adults	Although the mean number of medications concurrently prescribed per older adult decreased in the EO group at one year, the number significantly decreased across both groups (df = 3, F = 3.78, *p* < 0.02)
Avorn and Soumerai (1983): RCT (Chhina et al., 2013) [23]	Excessive prescribing of common medications (cephalexin, propoxyphene, and cerebral and peripheral vasodilators)	Pharmacist-led behavioural theory-based EO visits	The GPs in the EO group reduced the mean number of the common medications they prescribed (5439 mean units to 4174, *p* = 0.0001) 14% more than the GPs in the no-intervention control (5415 mean units to 4921) did at one year.
Berings, Blondeel, and Habraken (1994): RCT (Smith & Tett, 2010) [19]	Benzodiazepine prescribing	EO visit supplemented with mail-outs	Greater reductions in benzodiazepine prescribing was seen in GPs who received EO and mail-outs (21% between-group difference), as opposed to mail-outs alone (14% between-group difference)
Bernal-Delgado, Galeote-Mayor, Pradas-Arnal, and Peiro-Moreno (2002): RCT (Chhina et al., 2013) [23]	Anti-inflammatory prescribing	Structured EO with printed materials that explained tenoxicam was a less cost-effective option compared to diclofenac	Structured EO (Tenoxicam packages prescribed reduced by 22.5% [95%CI 34.42 to −10.76]) reduced tenoxicam prescribing significantly more than unstructured EO visits (Tenoxicam packages prescribed reduced by 9.78% [95%CI −17.70 to −1.86]).
Clyne (2014): RCT (Clyne et al., 2016) [2]	Inappropriate prescribing	Pharmacist-led EO, a GP-led medication review guided by web-based algorithms and information leaflets	Intervention group reduced inappropriate prescribing rates significantly more than usual care control group at one year (OR = 0.3, 95%CI = 0.1 to 0.7, *p* = 0.02)
De Burgh (1995): RCT (Chhina et al., 2013 [23]; Smith & Tett, 2010) [19]	Prescribing of benzodiazepines for insomnia and anxiety	Educational visit and supporting materials from a pharmacist or doctor	Intervention group reduced prescribing of benzodiazepines more than the control group, but this difference was not significant.
Gall, Harmer, and Wanstall (2001): before-and-after (Kamarudin et al., 2013) [27]	Inappropriate prescribing of supplements to malnourished patients	Practical and theoretical EO visit on how to use nutritional guidelines, assess for malnutrition and treat nutritional deficits	A significant 15% reduction in total prescribing of supplements was seen at three months (438 patients were prescribed supplements at baseline, which reduced to 372 patients at follow-up).
Graham, Hartzema, Sketris, and Winterstein (2008): cohort (Chhina et al., 2013) [23]	COX-2 prescribing	EO visit on evidence-based osteoarthritis management, emphasising minimising COX-2 prescribing.	General practitioners in intervention group significantly reduced COX-2 prescribing by 0.76 defined daily doses/patient compared to the control at 3 months; however, this effect was not maintained at 12 months.
Ilett et al. (2000): RCT (Chhina et al., 2013) [23]	Antibiotic prescribing	EO visit delivered by a therapeutics advisor involved delivering, and briefly discussing, the best practice guidelines for antibiotic prescription for otitis media, urinary tract infections, and upper and lower respiratory tract infections	The number of non-recommended antibiotic prescriptions (e.g. cefaclor and roxithromycin) per provider increased for GPs who received EO and GPs in the control group at three months. However, prescriptions of non-recommended antibiotics increased more for GPs in the control group, meaning non-recommended antibiotic prescribing decreased 74% more in the EO group.
Midlov, Bondesson, Eriksson, Nerbrand, and Hoglund (2006): RCT (Chhina et al., 2013 [23]; Kamarudin et al., 2013 [27]; Smith & Tett, 2010) [19]	Benzodiazepines prescribed to older adults	Two EO visits outlining the effects of long and medium acting benzodiazepines in older adults	General practitioners in the intervention group prescribed total (26.63%), and long and medium-acting benzodiazepines (25.80%) significantly less after one year compared to GPs in a wait-listed control group.
Peterson, Bergin, Nelson, and Stanton (1996): cohort (Chhina et al., 2013) [23]	Anti-inflammatory prescribing	EO program that emphasised reducing NSAID prescriptions mainly due to their negative side effects, and increasing use of other medications such as paracetamol, for people with rheumatic disease	Anti-inflammatory prescribing reduced by GPs in both intervention and control groups.
Ray et al. (2001): RCT (Chhina et al., 2013) [23]	Anti-inflammatory prescribing	EO program that emphasised reducing NSAID prescriptions mainly due to their negative side effects, and increasing use of other medications such as paracetamol, for people with osteoarthritis	EO, together with prompts to review NSAID prescription in patient files, significantly reduced the number of days patients had NSAIDs dispensed each year (between-group difference 7% [95%CI 3 to 11%]) by GPs. However, reductions in prescribing were seen in both groups.
Rognstad, Brekke, Fetveit, Dalen, and Straand (2013): RCT (Clyne et al., 2016) [2]	Inappropriate prescribing	GP-led EO program plus audit and feedback	The GPs in the intervention group (*n* = 250) reduced their inappropriate prescribing practices (measured using Beer’s criteria) by 12.1% (95%CI 16.8 to 6.9%) per 100 patients compared to GPs in the control group (education on antibiotic prescribing for respiratory infections)
Simon et al. (2006): RCT (Clyne et al., 2016) [2]	Inappropriate prescribing	Group EO program designed to increase acceptance of evidence-based computer alerts and was delivered alongside the integration of age-specific medication alerts that appear when potentially inappropriate medications (e.g. long-acting benzodiazepine) were entered by a GP into the patient’s medical record.	Adding EO to alerts did not enhance the efficacy of the alerts (which were also received by control group) at reducing inappropriate prescribing by GPs, as inappropriate prescriptions per 10,000 older adults decreased similarly for both groups (*p* = 0.52)
Tomson, Hasselström, Tomson, and Åberg (1997): RCT (Chhina et al., 2013) [23]	Prescribing of beta-2-agonists for asthma management	Tailored EO delivered twice per year and including oral and written information about evidence-based management of asthma	General practitioners in the intervention group significantly reduced their prescribing of beta-2-agonists and increased the prescribing of inhaled steroids but the between-group findings were not statistically significant
van Eijk, Avorn, Porsius, and de Boer (2001): RCT (Chhina et al., 2013) [23]	Prescribing anticholinergics to older adults	EO visits (individual vs. group) on the difficulties of managing anticholinergic side effects in older adults	The amount of highly anticholinergic antidepressants prescribed to older adults reduced by 26% (95% CI: - 4 to 48%) in the individual EO group and by 45% (95% CI: 8 to 67%) in the group EO group, compared to control groups.
Witt, Knudsen, Ditlevsen, and Hollnagel (2004): RCT (Chhina et al., 2013) [23]	Prescribing of beta-2-agonists for asthma management	One EO visit that involved discussing an evidence-based asthma guideline and supporting GPs to use it	General practitioners in the intervention group did not significantly reduce beta-2-agonist prescribing or increase the prescribing of inhaled steroids. Although, the reduction in beta-2-agonist and increase in inhaled steroid prescription, was 2 and 7% greater, respectively, for the intervention group compared to the control group.
Zwar, Wolk, Gordon, and Sanson-Fisher (2000): RCT (Chhina et al., 2013 [23]; Smith & Tett, 2010) [19]	Benzodiazepine prescribing	A 20-min EO visit about benzodiazepine prescribing	General practitioners in the intervention group reduced their prescribing rate (per 100 patient encounters) of benzodiazepines for all indications, including sleep problems and anxiety, from 2.3 to 1.7; however, this reduction was like that seen in the control group (a change of 2.2 to 1.6) who received an intervention on an unrelated topic

Notes

^a^Full citation available from the parent review or upon request

*COX-2 cyclooxygenase*-*2, EO* educational outreach, *GP* general practitioner, *NSAID* nonsteroidal anti-inflammatory drug, *RCT* randomised controlled trial

##### Can EO be used in isolation to successfully change the prescribing behaviour of health professionals?

The efficacy of isolated EO programs at changing health professional behaviour was examined in three reviews of systematic reviews and four systematic reviews [21–27].

Two reviews examined the efficacy of isolated EO programs at changing general health professional behaviour, without having a focus on prescribing behaviours. Johnson and May [26] included 67 systematic reviews to compare the efficacy of education, action and monitoring (e.g. audit and feedback), persuasion and providing information at changing health professional behaviour. This review found that both EO and action and monitoring interventions are more efficacious than persuasion and providing information at changing health professional behaviour [26]. A systematic review of 15 primary studies also found that health professionals who received a tailored EO intervention (e.g. specifically targeting barriers to changing professional behaviour) were almost twice as likely to change their behaviour (e.g. follow clinical guidelines) compared to those who received EO without such tailoring [21]. These reviews indicate that tailoring or supplementing of EO programs appears more effective than isolated programs at changing general health professional behaviour.

Five reviews examined the efficacy of isolated EO programs at changing prescribing behaviours of health professionals [22–25, 27]. Educational outreach programs were identified as efficacious at reducing inappropriate medication use and prescription in care homes [24] and at changing prescribing behaviours [23, 25]. However, other interventions (e.g. personalized feedback letters) might be just as successful as EO [22, 27]. This is an important insight as personalised feedback letters may be less costly than EO [27].

##### Can EO be used in multifaceted programs to successfully change the prescribing behaviour of health professionals?

The efficacy of multifaceted programs, of which EO is a main component, at changing prescribing behaviours of health professionals was examined in three reviews of systematic reviews and six systematic reviews [2, 6, 20, 22, 25, 26, 28–30]. Four reviews specifically addressed the efficacy of multifaceted programs at reducing potentially inappropriate prescribing (PIP), such as reducing the prescribing of antipsychotics to older adults (which can be considered inappropriate) [2, 20, 28, 30].

Chauhan et al. [22] included high-quality systematic reviews and found that multifaceted programs that include more than one of EO, audit and feedback, patient-mediated interventions and reminders can successfully change prescribing practices in primary care settings. Similarly, Green et al. [25] reported that delivering EO together with passive strategies (e.g. providing information leaflets), while considering the context in which the behaviour occurs and the people who are expected to change their behaviour, might be more efficacious at changing prescribing behaviours than delivering EO alone. Ostini et al. [29] reviewed nine high quality primary studies and found that EO was more successful when delivered together with audit and feedback and other educational materials. Although these reviews indicate that multifaceted EO interventions show promise for changing health professional prescribing behaviours, more research is required to determine the best mix of interventions and the role of EO within this mix as these reviews found different combinations of interventions to be efficacious at changing prescribing behaviour.

Three of the four included reviews that focused on PIP were specific to older adults. Interventions including EO, web-based treatment-alternative suggestions and tailored prescribing information was reported to successfully reduce PIP to older adults [2]. Another review found multifaceted EO programs lead to significant reductions in PIP in care homes [28]. Multifaceted educational interventions were also found to be efficacious at reducing the prescribing of antipsychotics to older adults [30]. Finally, a review that was not specific to older adults found that multifaceted EO programs including educational materials, audit and feedback, system support and practice facilitation (coaching) were successful at changing health professional behaviour, including PIP [20]. Overall, the findings from these reviews suggest that multifaceted EO programs can be efficacious at reducing PIP.

#### Interview results

Three providers of EO from government pharmacy and health professional regulation bodies participated in individual one-on-one interviews of approximately 30-min in duration. Five themes related to facilitating the successful delivery of EO programs to health professionals emerged and were used to supplement the findings of the review.

#### Results synthesis

##### Proactive vs. reactive

Interviewees indicated that increased participation and engagement with EO is more often seen for GPs who are being approached reactively (e.g. after something has gone wrong and they are upset) compared to when they are approached proactively (e.g. providing EO for professional development purposes).

*“They [the GPs] are fairly distressed because they’ve got patients that have either died or we’ve had to say that they’re not allowed to prescribe in that manner.”*

It was suggested that participation in EO is perceived negatively as a regulatory activity by GPs, as opposed to a professional development opportunity. However, the attitude and receptiveness of GPs varies:

“It's probably 60% in the middle who have two or three really problematic patients who are receptive to their engagement with us. They are very grateful for what we do and there's 20% who hate our guts.”

Furthermore, interviewees suggested that promoting EO as a risk management opportunity, to prevent something bad from happening in the future, might make EO more attractive to GPs.

##### Relationships matter

Interviewees stated that the relationship between the GP, the EO facilitator and the regulatory body needs to be positive to enhance engagement with EO. Being able to relate to the EO facilitator (e.g. by the facilitator being a GP) might encourage GPs to perceive them as credible partners in delivering best practice, rather than a regulator enforcing rules. For this, it is important to have the support of professional colleges and senior clinical leaders that are independent of the GP.

“The GPs probably need a no-blame, non-practice colleague…who is knowledgeable and skilled in those areas to actually be a bouncing point to actually work through some of the issues and to facilitate the GP getting their head around what they're trying to do and what their strategy is going to be.”

##### Practical learning enhances engagement

Content of EO visits needs to be practical, skills-focused and engaging to facilitate participation and uptake, as opposed to didactic or lecture-based.

“… just talking about evidence, best practice, taking no account of their practical difficulties or reality…the reception has been quite poor.”

“The education needs to be much less about ‘this is what an opiate does to the body and this is the dosage you use’, it's more about ‘Right, so you realise you want to use opiates. So, how do you do it, how do you bring the patient with you?’”

Furthermore, interviewees reported that in-person EO facilitates relationship building and better allows for tailoring content to the individual compared to online education delivery.

*“Depending on the conversation, depending on what they are interested in, quite often we veer into other topics and that’s fine. As long as they get something out of it and feel the activity is worthwhile.”*

##### Barriers to GPs engaging with EO

Interviewees reported several potential barriers limiting GP engagement with EO. The time needed to participate in EO and complete all relevant administrative paperwork, instead of seeing patients, can act as a barrier to completely engaging with EO. In this case, some GPs use personal time to participate in EO.

*“I guess we’re lucky in that the GPs that do participate make the time and, a lot of the time, it just takes up their lunch time.”*

The time and resources needed to attend EO, especially for rural and regional GPs, can act as barriers to participation. Furthermore, negative attitudes associated with EO can also prevent engagement.

“…they don't think they need continued education or updates because they know it all.”

##### Facilitators to GPs engaging with EO

Interviewees reported that having credible organisations delivering EO might enhance engagement and participation in EO. Credible organisations or professionals are those who are involved in research (e.g. present at conferences) and deliver evidence-based information.

“They [GPs] do generally trust that it's not biased, which is why they make time for us...”

Being able to talk to the same person over time might act as a facilitator for GPs proactively reaching out for education and advice.

“They [GPs] feel that they've got somebody that they spoke to last week that they can now ring up, proactively to say, ‘You haven't talked to me about this patient, but I've got a concern. Can I talk you through my concerns and have you got any advice?’”

Using incentives, like professional development points or monetary compensation, can motivate GPs to participate in EO. However, motivation to attend might not mean the GP is also motivated to learn from the experience and apply new skills clinically. Clinicians might only attend to ‘tick a box’ rather than to learn.

“They will attend something potentially for no other reason, they get two credits toward their 50 for the year, or whatever they have to do. They're not necessarily engaged in the issue. They're doing it because it serves another purpose, as opposed to being generally interested in the topic.”

## Discussion

The evidence review identified 14 reviews with moderate to low risk of bias and found that EO delivered both as an isolated program or as part of a multifaceted program can be efficacious at changing prescribing behaviours. However, multifaceted EO programs were more comprehensively researched than isolated programs and review-level literature specific to GPs is scarce. The practice review supplemented the evidence review by describing the characteristics of successful GP-focused EO programs, suggesting the use of presenters and organisations who are credible and/or well-known to GPs; use of incentives; and making EO compulsory, focused on practical content and preventing adverse outcomes might enhance GP engagement in EO and program success.

The review findings are supported by related reviews that have also examined the efficacy of education (e.g. EO, educational meetings and non-specific education) at changing health professional behaviours [7, 31–34]. However, the findings of this evidence and practice review also suggest that multifaceted EO programs might be more successful than isolated EO programs, especially if they include components such as tailoring and feedback. This more complex approach to delivering education has also been suggested as more efficacious than isolated approaches in a systematic review on educational meetings and professional practice change [32]. Using active techniques (e.g. group EO) together with, as opposed to instead of, passive techniques (e.g. information leaflets and online programs) and ensuring the EO program is tailored to both the professional and the clinical context might enhance the success of EO programs [7, 35].

Although review-level evidence suggests that multifaceted EO programs show promise for changing prescribing behaviours of health professionals, as well as professional behaviours [26], the changes might only be small. A review found that EO visits (delivered in isolation or in multifaceted programs) have small effects on the prescribing behaviours of health professionals, and that increasing the number of visits is unlikely to increase efficacy [6]. However, small improvements in prescribing behaviours might be clinically significant and have large effects at the population level [6]. Therefore, although the effect size of EO programs can appear small, EO programs can have population-level impact, especially for issues with high prevalence of which inappropriate prescribing is one.

Providing feedback (e.g. personalised feedback letters or audit and feedback) was reported by several included reviews as enhancing the efficacy of EO at changing health professional behaviour [20, 27, 29]. The use of personalised feedback letters, together with a safe prescribing injunction, has significantly reduced the amount of morphine dispensed by 861 US clinicians [36]. However, personalised feedback letters, sent together with educational materials, only reduced benzodiazepine prescribing a small amount by 374 Canadian primary care physicians [37]. Thus, feedback might be a useful addition to EO for changing health professional behaviour, but the efficacy of feedback might differ between professional and clinical contexts. The ideal components to include in multifaceted EO programs for different professional and clinical contexts remains unclear.

Overcoming clinician barriers (e.g. geographical) and enhancing the facilitators (e.g. incentives) might encourage attendance at EO and overall engagement, which can enhance the success of EO [32]. The practice review findings are supported by a review that found interactive and practical content is more efficacious at changing behaviour than didactic learning [32]. Results from a large cluster RCT also support the finding that GPs might be more inclined to follow advice of more skilled, or relatable, EO facilitators [38].

The practice review also indicated that EO content must be perceived as relevant to the GP; including content that is useful to them. Relevancy of the educational material has also been addressed in a recent pilot of self-audit of methadone treatment administration, which found self-audit was well received by GPs, suggesting self-audit might be a useful reflective practice and a way to make education relevant and interesting to the clinician [39]. Incorporating these facilitators and strategies to overcome barriers into EO programs might result in more tailored, engaging and successful EO programs.

There are some strengths and limitations that should be considered when interpreting the findings of this evidence and practice review. Where appropriate, the evidence review emphasised findings from three reviews of systematic reviews [22, 25, 26] due to their relatively higher methodological quality compared to individual systematic or narrative reviews [40]. Although several high-quality reviews were included to inform the conclusions of this review, EO was implemented differently in each, making it difficult to draw conclusions on the efficacy of individual programs across multiple studies.

The evidence review adopted rapid review methodology, which was necessary to provide timely advice to a government department. However, using rapid reviews risks ‘data dilution’, as findings are based on already summarised data in systematic reviews, and potentially missing relevant information. Furthermore, the inconsistency of data reported in the included reviews and the ‘rapid’ nature of the study design necessitated the use of narrative synthesis rather than meta-analysis during the data analysis process. Although using a rapid review was appropriate and necessary for this project, systematic reviews remain the definitive unit of knowledge translation.

All reviews were given a numerical AMSTAR 2 score to allow for direct bias risk comparisons between reviews. This enabled consideration of review findings in the context of their methodological rigor. Although presenting a numerical score to represent the risk of bias of the included reviews is a common practice [41], this involved a minor modification to the intended use of AMSTAR 2 [16].

The initial focus of the evidence review was to identify review-level evidence demonstrating the efficacy of EO at changing prescribing behaviours of GPs. However, despite a comprehensive search process, few reviews were identified that specifically examined all three components. Thus, all included reviews were examined to find any primary studies that addressed all three components, allowing for a summary of primary studies to be tabulated and used to supplement the findings of the review. This additional work is beyond the scope of conventional rapid reviews and provided a more in-depth understanding of the topic.

The practice review was designed to provide some detail on how EO programs happen in Victoria, Australia, and explore the factors that are associated with success. Three participants were interviewed; thus saturation of data was unlikely to be reached. The findings of the practice review should not be treated as a definitive qualitative exploration but instead be interpreted as supplementing the findings of the evidence review.

Future GP EO programs focused on changing prescribing behaviours should be multifaceted, including components that might enhance success (e.g. facilitated by a credible source and includes evidence-based education delivered in a face-to-face and practical learning environment), and examine if contextual factors (such as the demographics of the participant group, the timing of the intervention, the drug class of interest and the geographical context) influence program efficacy. Acknowledging that this literature and practice review was not a comprehensive qualitative exploration of this topic, future qualitative investigations including GPs who have experienced EO for changing prescribing behaviours should be used to inform intervention design. Furthermore, future programs should be tested using a cluster RCT design to optimise study quality, reduce chance for cross-contamination between intervention and control groups, and support the detection of effect associated with the EO program. The outcomes of the EO program should be measured at both the clinician and patient level and clearly distinguish the population being examined. Including these design components would facilitate more robust understanding of the effectiveness of EO interventions.

Although EO programs appear to be acceptable to GPs [42], acceptability will likely be influenced by local factors (e.g. case load and political influence). Thus, the acceptability and feasibility of any EO program should also be measured in a pilot study prior to wider implementation. Finally, the content (i.e. isolated or multifaceted) of the EO program should be accurately reported (i.e. by clearly explaining the strategies, such as feedback, provided and how it was delivered) using universally recognised terminology. Poor reporting limits the ability to identify and replicate the intervention components [43, 44].

## Conclusions

This evidence and practice review aimed to identify the efficacy of EO at changing prescribing behaviours of GPs and the factors that can enhance the success of EO. In conclusion, EO can be efficacious at changing health professional behaviour, including prescribing behaviours. The evidence to support the efficacy of EO at changing GP prescribing behaviour appears promising, although more research is needed. Based on interview findings, making EO programs practical, clinically relevant and using credible sources to deliver the program might enhance the success of EO programs at changing GP prescribing behaviour.

## Additional files

Additional file 1: Search Strategy. (DOCX 13 kb)

Additional file 2: Interview guide. (DOCX 13 kb)

## Data Availability

We declare that the data supporting the findings of this study (e.g. search strategy, characteristics of included studies and PRISMA statement) are available within the paper and its additional files. The datasets used and/or analysed during the current study are available from the corresponding author on reasonable request and will be provided only where ethical boundaries are not broken.

## Abbreviations

AMSTAR 2: A Measurement Tool to Assess Systematic Reviews 2
EO: Educational outreach
EPOC: Cochrane Effective Practice and Organisation of Care
GP: General practitioner
PIP: Potentially inappropriate prescribing
