# Supplementary material for: Changing prescribing behaviours with educational outreach: an overview of evidence and practice
Source: BMC Med Educ. 2019 Aug 14;19:311. doi: 10.1186/s12909-019-1735-3 (PMC6693161; doi:10.1186/s12909-019-1735-3)
Supplement: Supplementary file 1 — Search Strategy. (DOCX 13 kb) [file 12909_2019_1735_MOESM1_ESM.docx]

**Additional file 1:** CINAHL (EBSCOhost) search

Title and Abstract: (analgesi* OR alprazolam OR Buprenorphine OR Codeine OR “competent prescription“ OR Dexamphetamine OR Fentanyl OR Flunitrazepam OR Hydromorphone OR Ketamine OR Lisdexamfetamine OR methadone OR Methylphenidate OR Morphine OR Nabiximols OR opioid OR Oxycodone OR Pethidine OR prescri* OR “prescribing behavio?r” OR “prescription error” OR “prescription guideline” OR psychostimulant OR “safe prescription” OR “scheduled medication” OR “schedule 8” OR Sodium Oxybate OR Tapentadol)

AND

Title and Abstract: adher* OR adverse OR “?appropriate prescribing” OR “clinical competence” OR competen* OR complian* OR “decision making” OR drug OR “drug therapy” OR error OR “guideline adherence” OR “health professional performance” OR “irrational prescribing” OR judgement OR knowledge OR medication OR “professional practice” OR “quality assurance” OR risk OR “standards of practice” OR safe OR skill

AND

MESH: inappropriate prescribing OR drug prescriptions

AND

Title and Abstract: doctor OR “family doctor” OR “general practitioner” OR GP

AND

Title and Abstract: “clinical practice” OR “community health care” OR “primary care” OR “primary care practitioner”

AND

MESH: general practitioners

AND

Title and Abstract: “academic detailing” OR education OR “educational outreach visit” OR “educational meeting” OR “educational visit” OR intervention OR “knowledge translation” OR lecture OR material OR meeting OR “professional development” OR training OR “university-based educational detailing” OR workshop

AND

MESH: education, medical
