# Supplementary material for: Changing prescribing behaviours with educational outreach: an overview of evidence and practice
Source: BMC Med Educ. 2019 Aug 14;19:311. doi: 10.1186/s12909-019-1735-3 (PMC6693161; doi:10.1186/s12909-019-1735-3)
Supplement: Supplementary file 2 — Interview guide. (DOCX 13 kb) [file 12909_2019_1735_MOESM2_ESM.docx]

# **Additional file 2:** Interview guide

1. Can you provide a brief introduction and outline your experience in the area of educational outreach to encourage safe prescribing in general practice, including how long you have been in this role?

2. From your perspective and experience, what are key issues that need to be addressed to optimise educational outreach in this general practice?

3. What strategies are you aware of that have been employed in the past to promote educational outreach in general practice?

4. a) (if answered 3) How successful have these strategies been?

b) (if answered 4a) What factors do you think have contributed to the success or failure of these strategies?

5. Do you have any other comments in relation to educational outreach to encourage safe prescribing in general practice?
